# The Rhizome Mixture of *Anemarrhena asphodeloides* and *Coptidis chinensis* Ameliorates Acute and Chronic Colitis in Mice by Inhibiting the Binding of Lipopolysaccharide to TLR4 and IRAK1 Phosphorylation

**DOI:** 10.1155/2014/809083

**Published:** 2014-09-03

**Authors:** Jin-Ju Jeong, Se-Eun Jang, Supriya R. Hyam, Myung Joo Han, Dong-Hyun Kim

**Affiliations:** ^1^Department of Life and Nanopharmaceutical Sciences and Department of Pharmacy, College of Pharmacy, Kyung Hee University, 1 Hoegi, Dongdaemun-Gu, Seoul 130-701, Republic of Korea; ^2^Department of Food and Nutrition, Kyung Hee University, 1 Hoegi, Dongdaemun-Gu, Seoul 130-701, Republic of Korea

## Abstract

In the previous study, the mixture of the rhizome of *Anemarrhena asphodeloides* (AA, family Liliaceae) and the rhizome of *Coptidis chinensis* (CC, family Ranunculaceae) (AC-mix) improved TNBS- or oxazolone-induced colitis in mice. Therefore, to investigate its anticolitic mechanism, we measured its effect in acute and chronic DSS-induced colitic mice and investigated its anti-inflammatory mechanism in peritoneal macrophages. AC-mix potently suppressed DSS-induced body weight loss, colon shortening, myeloperoxidase activity, and TNF-α, IL-1*β*, and IL-6 expressions in acute or chronic DSS-stimulated colitic mice. Among AC-mix ingredients, AA, CC, and their main constituents mangiferin and berberine potently inhibited the expression of proinflammatory cytokines TNF-α and IL-1*β*, as well as the activation of NF-*κ*B in LPS-stimulated peritoneal macrophages. AA and mangiferin potently inhibited IRAK phosphorylation, but CC and berberine potently inhibited the binding of LPS to TLR4 on macrophages, as well as the phosphorylation of IRAK1. AC-mix potently inhibited IRAK phosphorylation and LPS binding to TLR4 on macrophages. Based on these findings, AC-mix may ameliorate colitis by the synergistic inhibition of IRAK phosphorylation and LPS binding to TLR4 on macrophages.

## 1. Introduction

Inflammatory bowel diseases (IBD), including ulcerative colitis and Crohn's disease, is a severe form of intestinal inflammation [1]. Its pathogenic mechanism is assumed to be attributed to complex mucosal immune responses to antigens, such as resident enteric bacterial toxins, in the gastrointestinal tract. These bacterial endotoxins, such as lipopolysaccharide (LPS), stimulate the mucosal immune system and induce proinflammatory cytokines and other mediators, activating the inflammatory reaction of the immune system via canonical and noncanonical NF-*κ*B signaling pathways through toll-like receptors (TLRs) and/or cytokine receptors [2–4]. TLRs are a class of protein that plays a key role in the innate and adaptive mucosal immune defense systems against pathogens. Among TLRs, TLR4 recognizes LPS and initiates a signaling cascade through the Toll/IL-1R (TIR) domain of its cytoplasmic tail and myeloid differentiation factor (MyD)88, allowing for subsequent activation of IL-1R-associated kinases (IRAKs). All IRAK members form multimeric receptor complexes. Phosphorylated IRAK-1 activates a multimeric protein complex (TRAF6/TAK1/TAB1/TAB2), leading to the activation of NF-*κ*B as well as the expression of proinflammatory cytokines. Therefore, to cure inflammatory diseases, natural products regulating NF-*κ*B signaling pathway have been attempted [5, 6].

The rhizome mixture of* Anemarrhena asphodeloides* (AA, family Liliaceae) and the rhizome of* Coptidis chinensis *(CC, family Ranunculaceae) (AC-mix) exhibits anti-inflammatory effects in TNBS- or oxazolone-induced colitic mice [7]. Of these ingredients, AA has been used as an antipyretic, antiphlogistic, sedative, and diuretic agent and CC has been used to treat patients who have gastroenteritis and diarrhea in tradition Chinese medicine [8–10]. AA contains saponins such as timosaponin AIII and xanthones such as mangiferin [11]. CC contains isoquinoline alkaloids such as berberine. Of these constituents, mangiferin inhibits lung inflammation and pruritus in mice [12, 13] and LPS-induced NF-*κ*B signal pathway in macrophages [14]. Berberine exhibits bacteriocidal, anticholera toxin, cholesterol-lowering, anti-inflammatory, and anticolitic effects [15–18]. Berberine also inhibits TNBS-induced colitis in mice [10, 15]. Nevertheless, the anticolitic mechanism of AC-mix has not been studied thoroughly.

Therefore, we investigated the anticolitic effect of AC-mix in dextran sulfate sodium- (DSS-) induced colitic mice and its anti-inflammatory mechanism in LPS-stimulated peritoneal macrophages.

## 2. Materials and Methods

### 2.1. Chemicals and Reagents

RPMI1640, DSS, hexadecyl trimethyl ammonium bromide, and radio-immunoprecipitation assay (RIPA) lysis buffer were purchased from Sigma (St Louis, MO, USA). The protease inhibitor cocktail was purchased from Roche Applied Science (Mannheim, Germany). Enzyme-linked immunosorbent assay (ELISA) kits were from Pierce Biotechnology, Inc. (Rockford, IL, USA). Antibodies were purchased from Cell Signaling (Cell Signaling Technology, Inc. (Danvers, MA, USA). The enhanced chemiluminescence (ECL) immunoblot system was purchased from Pierce Co. (Rockford, IL, USA). Mangiferin and berberine were isolated from the previously reported methods of Lee et al. [10] and [13], respectively.

### 2.2. Extraction

Extracts of herbal medicines, AA and CC rhizomes, were prepared according to the previously reported method of Paradkar et al. [5]. Briefly, each pulverized herbal medicine (100 g) was extracted three times with 80% EtOH under water bath. The 80% EtOH extracts were combined and evaporated to dryness under reduced pressure (yield of AA, 24%; yield of CC, 21%). AC-mix was the mixture of AA and CC (=1 : 1). The contents of mangiferin and berberine in AC- mix were 0.31% and 2.02%, respectively.

### 2.3. Animals

Male C57BL/6 (20–23 g, 6 weeks) were supplied from the Central Animal Breeding Center (Seoul, Korea). All animals were housed in wire cages at 20–22°C and 50 ± 10% humidity, fed standard laboratory chow (Samyang Co., Seoul, Korea), and allowed water ad libitum. All procedures relating to animals and their care conformed to the international guidelines “Principles of Laboratory Animals Care” (Kyung Hee University, animal experiment guideline 2006 and NIH publication number 85-23 revised 1985).

### 2.4. Isolation and Culture of Peritoneal Macrophages

Male C57BL/6 mice were intraperitoneally injected with 2 mL of 4% sodium thioglycolate solution [6]. Mice were sacrificed on the 4th day after the injection of sodium thioglycolate and the peritoneal cavities were flushed with 10 mL of RPMI 1640. The peritoneal lavage fluids were centrifuged at 200 ×g for 10 min, resuspended with RPMI 1640, distributed in 12-well plates, incubated for 1 h at 37°C, and washed twice. Nonadherent cells were removed by aspiration. The attached cells (0.5 × 10^6^ cells/well) were cultured at 37°C in RPMI 1640 plus 10% FBS. The cells were used as peritoneal macrophages. To examine the anti-inflammatory effect of test agents, peritoneal macrophages were incubated in the absence or presence of test agents with 100 ng/mL LPS. The cytotoxicities of these agents in the cell viability were measured using the trypan blue staining.

### 2.5. Preparation of DSS-Induced Colitis in Mice

First, DSS-induced acute colitis was induced in mice with 3% DSS (dissolved in drinking water) for 7 days [19]. Normal control mice were given drinking water alone. Test agents (AC-mix, 10 and 20 mg/kg; mesalazine, 10 mg/kg dissolved in 2% tween 80) were administered via oral gavage once a day for 3 days after the final DSS administration. The mice were killed 20 h after the final administration of test agents.

Second, DSS-induced chronic colitis was induced in mice by alternative administration of drinking waters with or without 2.5% DSS [20]. First cycle was for 14 days. In days 1–7, drinking water with DSS was supplied to the mice and then replaced with drinking water without DSS for next 7 days (days 8–14). The second cycle was for 10 days. In days15–19 drinking water with DSS was allowed and then in days 20–24 water without DSS was given. The third cycle was for 6 days. In days 25–27 drinking water with DSS was given and then in days 28–30 drinking water without DSS was given. Normal mice were given drinking water for 30 days.

Test agents (AC, 10 and 20 mg/kg; MS, 10 mg/kg; mesalazine, 20 mg/kg dissolved in 2% tween 80) were administered via oral gavage once a day for 15 days from day 16. The mice were killed on day 31.

The colons of the killed mice were quickly removed, opened longitudinally, and gently cleared of stool by PBS [6]. Macroscopic assessment of the disease grade was scored according to a previously reported scoring system (0, no ulceration and no inflammation; 1, no ulceration and local hyperemia; 2, ulceration without hyperemia; 3, ulceration and inflammation at one site only; 4, two or more sites of ulceration and inflammation; 5, ulceration extending more than 2 cm), and the colon tissue was then used for ELISA and immunoblotting [6].

### 2.6. Colon Tissue Preparation

The mice's colons were excised and perfused with ice-cold perfusion solution (0.15 M KCl, 2 mM EDTA, pH 7.4), like the previously reported [21] and were homogenized in ice-cold RIPA lysis buffer (1 mL/intestine wet weight g) containing 1% phosphatase inhibitor cocktail and 1% protease inhibitor cocktail at 4°C for 10 min. The lysate was centrifuged at 15,000 ×g and 4°C for 10 min, and the resulting supernatant was used for myeloperoxidase activity, immunoblotting, and ELISA.

### 2.7. Assay of Myeloperoxidase Activity

An aliquot (50 *μ*L) of the supernatant of the colon homogenate was added to a reaction mixture of 1.6 mM tetramethylbenzidine and 0.1 mM H_2_O_2_ and incubated at 37°C [21]. And then the absorbance was measured at 650 nm over time. Myeloperoxidase activity was defined as the quantity of enzyme degrading 1 *μ*mol/mL of peroxide at 37°C and expressed in unit/mg protein. The protein content was assayed by the method of Bradford.

### 2.8. ELISA and Immunoblotting

For the ELISA of TNF-α, IL-1*β*, IL-6, and IL-10, the colon homogenate or the macrophage-cultured supernatants were transferred to 96-well ELISA plates. TNF-α, IL-1*β*, IL-6, and IL-10 concentrations were assayed using commercial ELISA kits (Pierce Biotechnology, Inc., Rockford, IL, USA) [21].

For the immunoblot analyses of p-IRAK1, p-IKK*β*, p-p65, and *β*-actin, the colon homogenate or the macrophage homogenate supernatants were used for the immunoblotting. The supernatants were subjected to electrophoresis on 8–10% sodium dodecyl sulfate-polyacrylamide gel and then transferred to nitrocellulose membrane. Levels of p-IRAK1, p-IKK*β*, p-p65, and *β*-actin were assayed as previously described [21]. Immunodetection was carried out using an enhanced chemiluminescence detection kit.

### 2.9. Immunofluorescent Confocal Microscopy

For the LPS-TLR4 complex assay, the peritoneal macrophages were plated on cover slides and incubated at 37°C for 20 h. The cells were stimulated with Alexa Fluor 594-conjugated LPS (10 *μ*g/mL) for 20 min in the absence or presence of AC-mix, AA, CC, mangiferin, or berberine. The cells were fixed with 4% paraformaldehyde and 3% sucrose for 20 min, stained with rabbit polyclonal anti-TLR4 antibody for 2 h at 4°C, and incubated with Alexa Fluor 488-conjugated secondary antibodies for 1 h and then assessed by a confocal microscope.

### 2.10. Flow Cytometry

The peritoneal macrophages were treated with Alexa Fluor 488-conjugated LPS (10 *μ*g/mL) for 10 min. The cells were fixed in PBS containing 3% sucrose and 4% paraformaldehyde for 20 min and washed with PBS and then incubated with propidium iodide (10 *μ*g/mL) for 10 min and measured by a flow cytometer (C6 Flow Cytometer System).

### 2.11. Statistical Analysis

All data are expressed as the mean ± standard deviation (S.D.), with statistical significance analyzed using one-way ANOVA followed by a Student-Newman-Keuls test.

## 3. Results

### 3.1. Inhibitory Effect of AC-Mix on DSS-Induced Colitis in Mice

**Figure 1:**
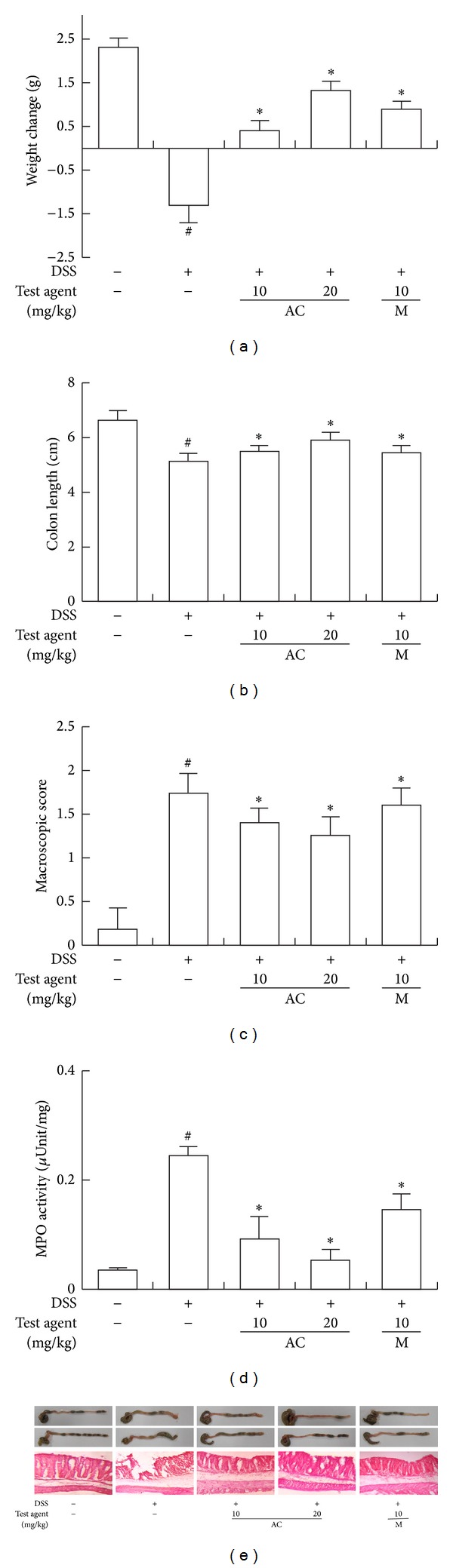
Effect of AC-mix on body weight loss (a), colon length (b), macroscopic score (c), myeloperoxidase (MPO) (d), and colon histology (e) in DSS-induced acute colitic mice. DSS, except in the control group, was orally administered with the drinking water to mice treated with saline, AC-mix, or mesalazine for 7 days. Test compounds [AC-mix (AC, 10 and 20 mg/kg), mesalazine (M, 10 mg/kg), or saline] were orally administered for 3 days after DSS treatment. The mice were sacrificed at 20 h after the final administration of test agents. All values are the mean ± S.D. (*n* = 6). ^#^ *P* < 0.05 versus normal control group; *P < 0.05 versus DSS group.

**Figure 2:**
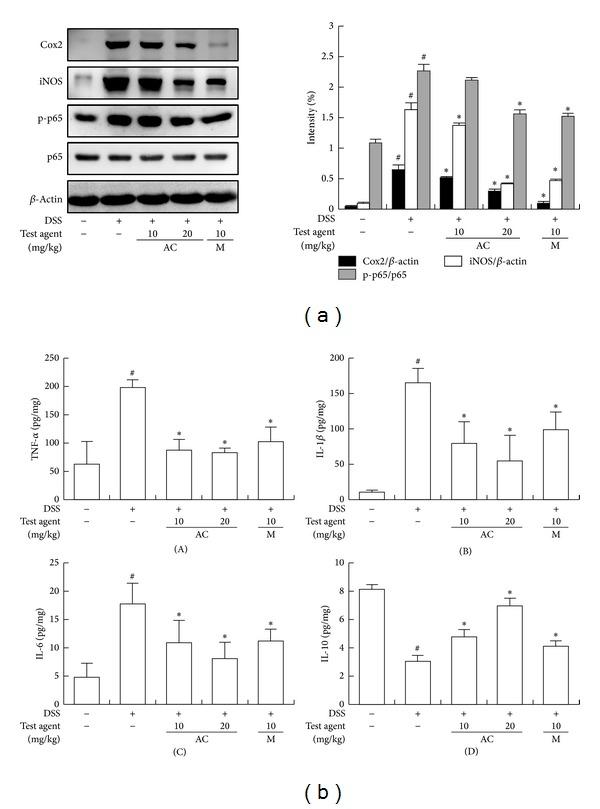
Effect of AC-mix on the activation of NF-*κ*B and the expression of COX-2, iNOS, TNF-α, IL-1*β*, IL-6, and IL-10 in DSS-induced acute colitic mice. DSS, except in the control group, was orally administered with the drinking water to mice treated with saline, AC-mix, or mesalazine for 7 days. Test compounds [AC-mix (AC, 10 and 20 mg/kg), mesalazine (M, 10 mg/kg), or saline] were orally administered for 3 days after DSS treatment. The mice were sacrificed at 20 h after the final administration of test agents. (a) Effect on the activation of NF-*κ*B and the expression of COX-2, iNOS, and *β*-actin. The proteins were analyzed by immunoblotting. (b) Effect on the levels of TNF-α (A), IL-1*β* (B), IL-6 (C), and IL-10 (D). These cytokines were analyzed by ELISA kits. All values are the mean ± S.D. (*n* = 6). ^#^ *P* < 0.05 versus normal control group; *P < 0.05 versus DSS group.

First, we measured anti-inflammatory effect of AC-mix on DSS-induced acute colitis in mice. DSS caused body weight loss and colon shortening to 93.1% and 76.8% of normal control mice, respectively, and increased myeloperoxidase activity 7.4-fold compared to that of normal control mice (Figure 1). Treatment with AC-mix inhibited DSS-induced body weight loss, colon shortening, and myeloperoxidase activity. AC-mix (20 mg/kg) also inhibited the myeloperoxidase activity by 89.5%, compared with the DSS-treated group (*P* < 0.05). DSS treatment also induced iNOS and COX-2 expression and activated NF-*κ*B (Figure 2). However, AC-mix inhibitied the expression of iNOS and COX-2 in acute DSS-induced colitis, as well as the activation of NF-*κ*B. AC-mix (20 mg/kg) suppressed DSS-induced TNF-α, IL-1*β*, and IL-6 expressions by 84.7%, 71.2%, and 73.2%, respectively. However, AC-mix upregulated IL-10 expression suppressed by DSS treatment. Anticolitic effect of AC-mix was comparable to that of mesalazine.

**Figure 3:**
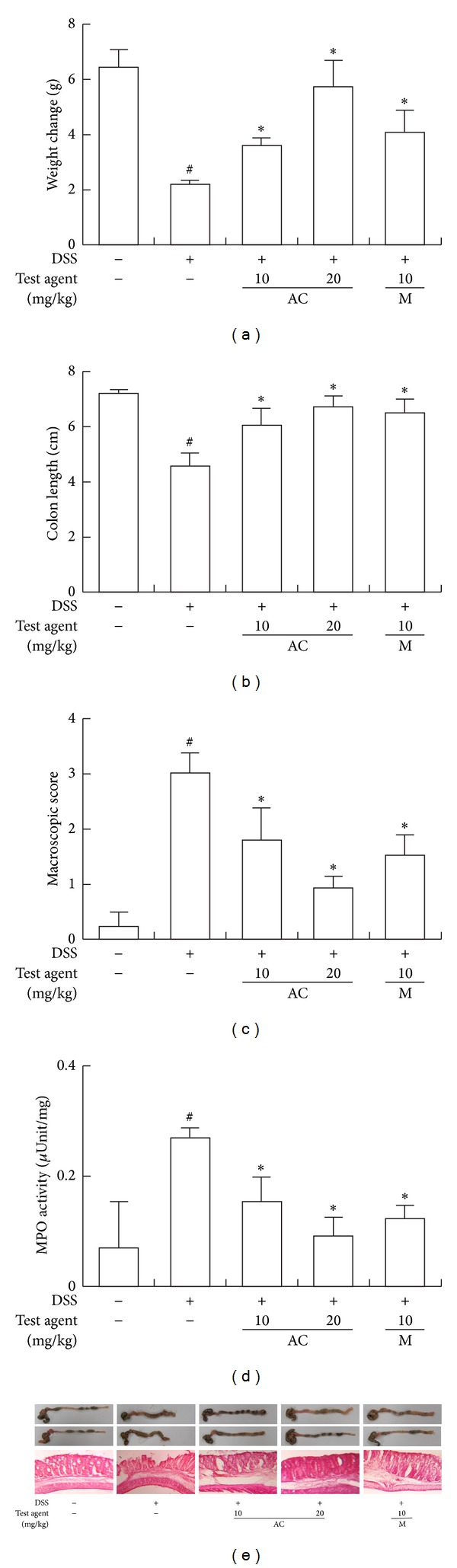
Effect of AC-mix on body weight loss (a), colon length (b), macroscopic score (c), myeloperoxidase (MPO) (d), and histology (e) in DSS-induced chronic colitic mice. DSS, except in the control group, was orally administered with the drinking water to mice treated with saline, AC-mix, or mesalazine, as indicated in Materials and Methods. Test compounds [AC-mix (AC, 10 and 20 mg/kg), mesalazine (M, 10 mg/kg), or saline] were orally administered for 15 days after DSS treatment. The mice were sacrificed at 20 h after the final administration of test agents. All values are the mean ± S.D. (*n* = 6). ^#^ *P* < 0.05 versus normal control group; *P < 0.05 versus DSS group.

**Figure 4:**
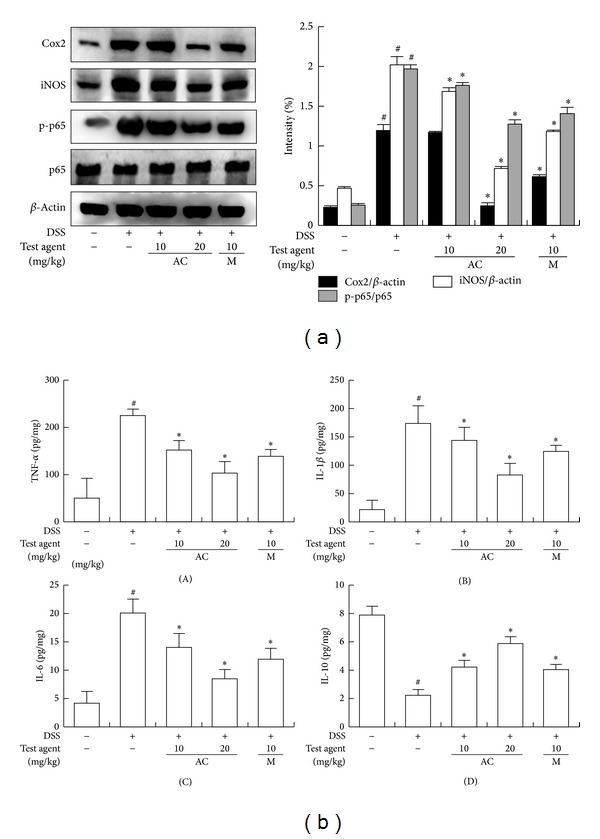
Effect of AC-mix on the activation of NF-*κ*B and the expression of COX-2, iNOS, TNF-α, IL-1*β*, IL-6, and IL-10 in DSS-induced acute colitic mice. DSS, except in the control group, was orally administered with the drinking water to mice treated with saline, AC mix or mesalazine, as indicated in Materials and Methods. Test compounds [AC-mix (AC, 10 and 20 mg/kg), mesalazine (M, 10 mg/kg), or saline] were orally administered for 15 days after DSS treatment. The mice were sacrificed at 20 h after the final administration of test agents. (a) Effect on the activation of NF-*κ*B and the expression of COX-2, iNOS, and *β*-actin. The proteins were analyzed by immunoblotting. (b) Effect on the levels of TNF-α (A), IL-1*β* (B), IL-6 (C), and IL-10 (D). These cytokines were analyzed by ELISA kits. All values are the mean ± S.D. (*n* = 6). ^#^ *P* < 0.05 versus normal control group; *P < 0.05 versus DSS group.

Next, we measured the inhibitory effect of AC-mix in DSS-induced chronic colitic mice (Figure 3). Chronic treatment with DSS caused body weight loss and severe colon shortening, manifested with severe edema, and increased myeloperoxidase activity (Figure 4). Treatment with AC-mix (20 mg/kg) significantly inhibited DSS-induced body weight loss, colon shortening, and myeloperoxidase activity by 81.2% and 73.3%, respectively, compared with the DSS-treated group (*P* < 0.05). Chronic treatment with DSS also induced the expression of iNOS and COX-2 and the activation of NF-*κ*B (Figure 4). However, AC-mix inhibited DSS-induced iNOS and COX-2 expressions, as well as NF-*κ*B activation. AC-mix treatment also inhibited DSS-induced TNF-α, IL-1*β*, and IL-6 expressions but increased the IL-10 expression. Anticolitic effect of AC-mix was comparable to that of mesalazine.

### 3.2. Inhibitory Effect of AC-Mix, AA, CC, Mangiferin, and Berberine on IRAK1 Phosphorylation and NF-*κ*B Activation in LPS-Stimulated Peritoneal Macrophages

**Figure 5:**
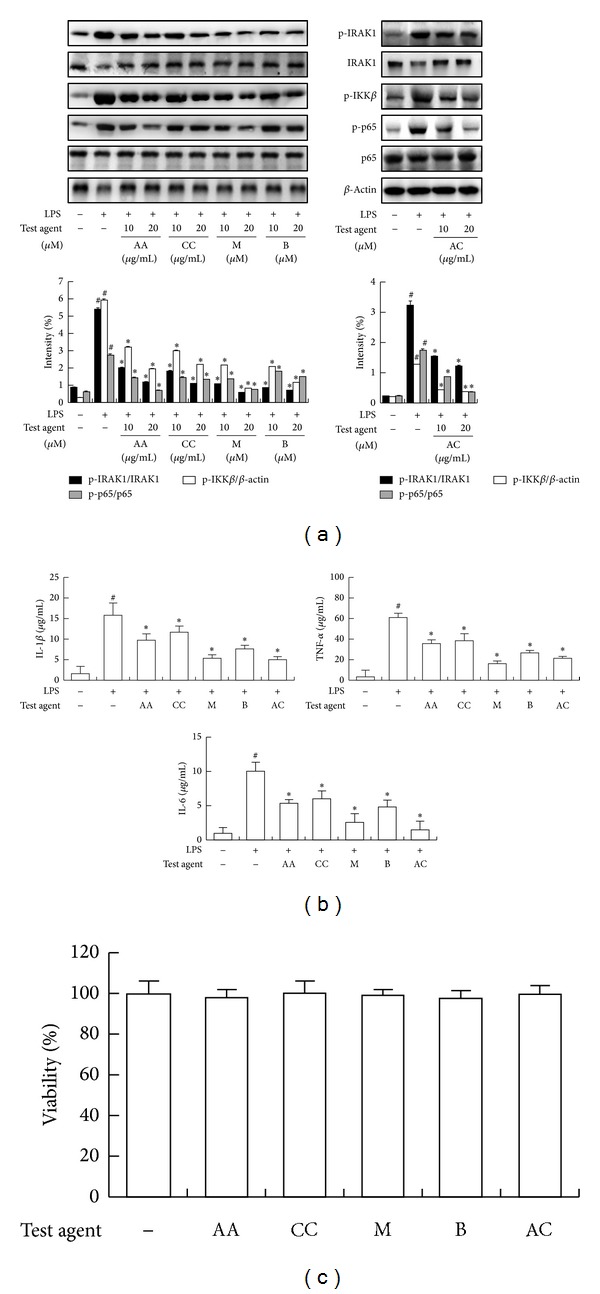
Inhibitory effect of AC-mix, AA, CC, mangiferin, and berberine on the activation of IRAK1, IKK*β*, and NF-*κ*B and the expression of TNF-α, IL-1*β*, and IL-6 in LPS-stimulated peritoneal macrophages. (a) Effect on the activation of IRAK1, IKK*β*, and NF-*κ*B. Test agents were treated for 90 min in LPS-stimulated macrophages and then the proteins were measured by immunoblotting. (b) Effect on the expression of TNF-α, IL-1*β*, and IL-6. Test agents (AC, AA, CC, mangiferin and berberine) were treated for 24 h min in LPS-stimulated macrophages and then the cytokines were measured by ELISA. (c) Cell viability, measured by tryphan blue staining assay. Test agents [AC-mix, 20 *μ*g/mL; AA, 20 *μ*g/mL; CC, 20 *μ*g/mL; mangiferin, 20 *μ*M; and berberine, 20 *μ*M] were treated for 2 days. All data are expressed as mean ± S.D. (*n* = 4 in a single experiment). *Significantly different versus group treated with LPS alone (*P* < 0.05). ^#^Significantly different versus LPS-nontreated group (*P* < 0.05).

Next, we tested the ability of AC-mix to inhibit NF-*κ*B activation in LPS-induced peritoneal macrophages (Figure 5(a)). Exposure to LPS activated NF-*κ*B and increased IRAK1 and IKK*β* phosphorylation. Treatment with AC-mix suppressed LPS-induced IRAK1 and IKK*β* phosphorylation and NF-*κ*B activation. Therefore, we investigated the effect of AC ingredients AA, CC, mangiferin, and berberine on the phosphorylation of IRAK and IKK*β* in LPS-stimulated peritoneal macrophages (Figure 5(b)). Treatment with LPS significantly induced the phosphorylation of IRAK1 and IKK*β*, but reduced IRAK1 expression. However, treatment with AC-mix ingredients significantly inhibited LPS-induced IKK*β* and IRAK1 phosphorylation and IRAK1 reduction. Of them, mangiferin inhibited IKK*β* and IRAK1 phosphorylation and NF-*κ*B activation most potently, followed by berberine. Mangiferin and berberine also potently inhibited LPS-induced TNF-α, IL-1*β*, and IL-6 expressions, like the previously reported. To determine whether AC, AA, CC, mangiferin, and berberine were cytotoxic, peritoneal macrophages were treated with AC (20 *μ*g/mL), AA (20 *μ*g/mL), BB (20 *μ*g/mL), mangiferin (20 *μ*M), and berberine (20 *μ*M) for 48 h. No cytotoxic effect of tested agents was observed under the experimental conditions (Figure 5(c)).

**Figure 6:**
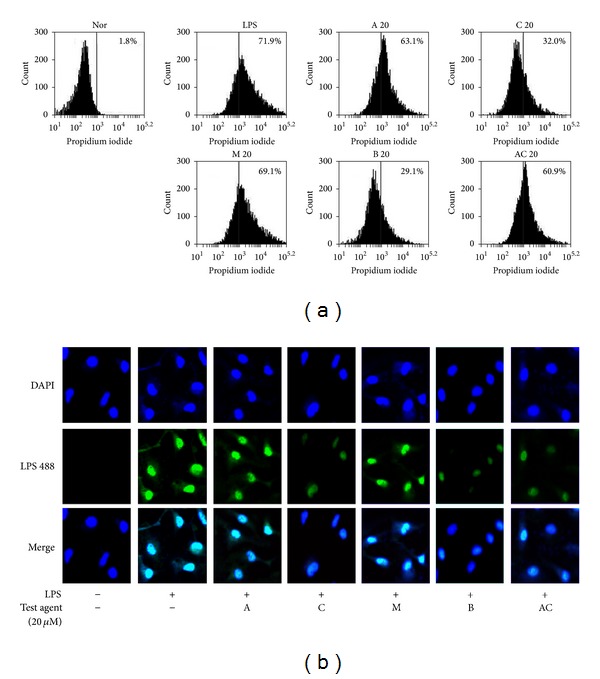
Effect of AC-mix, AA, CC, mangiferin, and berberine on the binding of LPS to TLR-4 on peritoneal macrophages. The macrophages isolated from mice were incubated with Alexa Fluor 488-conjugated LPS for 20 min in the absence or presence of AC-mix (20 *μ*g/mL), AA (20 *μ*g/mL), CC (20 *μ*g/mL), mangiferin (20 *μ*M), and berberine (20 *μ*M) and then analyzed by FACS (a) and confocal microscope (b).

Therefore, to investigate the anti-inflammatory target molecules of AC-mix, we also examined the ability of AC-mix and its ingredients to inhibit the interaction of LPS to TLR4 in the peritoneal macrophages using flow cytometry and confocal microscopy analyses. When the peritoneal macrophages were incubated with Alexa Fluor 488-conjugated LPS alone, LPS-bound macrophages were significantly shifted by flow cytometry analysis (Figure 6(a)). Treatment with AC, CC, and berberine inhibited the shift of macrophages by Alexa Fluor 488-conjugated LPS. Furthermore, AC, CC, and berberine inhibited the binding of Alexa Fluor 488-conjugated LPS to the macrophages by confocal microscope analysis (Figure 6(b)). However, AA and mangiferin did not inhibit the binding of LPS to macrophages by flow cytometry and confocal microscopy analyses.

## 4. Discussion

IBD is a complex disease caused by genetic and environmental factors, such as pathogens [22, 23]. The alteration of gut microbiota by diets or medications could stimulate the innate immune system through pattern recognition receptors [24]. Among these, TLR4 is highly expressed in the colons of IBD patients [2]. TLR4 is a pattern recognition molecules receptor for LPS [4, 24, 25]. LPS interacts with circulating LPS-binding protein and binds to TLR4 on the cell membrane with two coreceptors CD14 and myeloid differentiation protein (MD)2, then activating MyD88-dependent and MyD88-independent TLR4 signaling pathways. The MyD88-dependent pathway activates NF-*κ*B and AP-1 through the activation of IKK and MAPKs, respectively, regulating the expression of proinflammatory cytokines TNF-α and IL-1*β*. Thus, the stimulation of LPS activates the secretion of proinflammatory cytokines from monocytes, macrophages, and dendritic cells, leading to inflammation. These proinflammatory cytokines TNF-α and IL-1*β* are activated through canonical and noncanonical NF-*κ*B, which is a heterodimer consisting of five members, including RelA (p65), cRel, RelB, NF-*κ*B1 p50, and NF-*κ*B2 p52 [26, 27]. Canonical NF-*κ*B is consisted of mainly p65/p50 and noncanonical one is of RelB/p52. The former is mainly involved in natural immunity and most inflammations, while the latter is in B-cell maturation and autoimmune diseases. NF-*κ*B is an essential transcription factor for proper immune functions, but its excess activation often causes inflammation [28]. Therefore, to cure IBD, natural products inhibiting canonical NF-*κ*B and MAPKs/AP-1 signal pathways have been screened [6].

In the previous study, we found that AC-mix potently inhibit TNBS- or oxazolone-induced colitis in mice [5]. AC-mix ingredients AA and CC did not exhibit toxicity such mortality and remarkable clinical signs in subchronic toxicity study [29, 30] and showed anti-inflammatory effects [7, 10]. In the present study, AC-mix also potently inhibited body weight loss, colon shortening, and myeloperoxidase increasing in mice acutely and chronically treated with DSS. AC-mix also suppressed the DSS-induced IRAK-1 phosphorylation and NF-*κ*B activation. Furthermore, AC-mix inhibited the DSS-induced expression of inflammatory markers, such as COX-2, iNOS, TNF-α, IL-1*β*, and IL-6. In in vitro study, AC-mix inhibited the expression of proinflammatory cytokines TNF-α, IL-1*β*, and IL-6, as well as the activation of NF-*κ*B in LPS-stimulated peritoneal macrophages. AC-mix also inhibited the expression of COX-2 and iNOS in LPS-induced peritoneal macrophages. AC-mix ingredients AA, CC, mangiferin, and berberine also inhibited the expression of proinflammatory cytokines TNF-α, IL-1*β*, and IL-6, as well as the activation of NF-*κ*B in LPS-stimulated peritoneal macrophages. Of these ingredients, AA and its main constituent mangiferin potently inhibited IRAK-1 phosphorylation in LPS-stimulated macrophages but did not inhibit the binding of LPS to TLR4 on the macrophages. However, CC and its main constituent berberine potently inhibited LPS binding to TLR4 on the macrophages, as well as IRAK-1 phosphorylation. In addition, berberine inhibits NF-*κ*B, Akt, and MAPK signaling pathways in LPS-stimulated THP-1 cells [29] and mangiferin inhibits PI3K and MAPK signaling pathways in PMA-stimulated human astroglioma cells [31]. These suggest that AA and mangiferin may inhibit inflammation by regulating NF-*κ*B and AP-1 activation via the inhibition of IRAK-1 phosphorylation, and CC and berberine may inhibit the inflammation by regulating NF-*κ*B and AP-1 activation via the inhibition of LPS binding to TLR4 on the macrophages. In the present study, we found that anti-inflammatory effect of AC-mix, which contains 0.31% mangiferin and 2.02% berberine, was comparable to those of mangiferin or berberine alone. These suggest that AC-mix may suppress the inflammation by synergistic anti-inflammatory actions of AA and CC constituents, particularly their constituents, mangiferin and berberine. It is supported by the previous reports that mangiferin inhibits LPS-induced NF-*κ*B signal pathway in macrophages [14] and berberine has been reported to exhibit bacteriocidal, anti-inflammatory, and anticolitic effects [8, 13–15]. Furthermore, thimosaponins, additional constituents of AA, also inhibit the expression of proinflammatory cytokines TNF-α and IL-1*β* in BV-2 cells and peritoneal macrophages [32].

Based on these findings, we insist that AC-mix may inhibit acute and chronic colitis by inhibiting NF-*κ*B activation and be one of the candidates for the treatment of IBD.
